# METHODS AND BASELINE CHARACTERISTICS FOR THE TECHSAGE TELE TAI CHI CLINICAL TRIAL

**DOI:** 10.1093/geroni/igad104.0126

**Published:** 2023-12-21

**Authors:** Tracy Mitzner, Kara Mumma, Elena Remillard

**Affiliations:** Georgia Institute of Technology, Atlanta, Georgia, United States; Georgia Institute of Technology, Atlanta, Georgia, United States; Georgia Institute of Technology, Atlanta, Georgia, United States

## Abstract

Individuals aging with long-term mobility disabilities encounter a range of significant barriers to participating in physical and social activities, from challenges with transportation, to lack of accessibility and access to appropriate instruction. We developed a Tai Chi telewellness intervention to facilitate both physical and social wellness through accessible, virtual group classes. The intervention translated an in-person, evidence-based tai chi program (Tai Chi for Health Institute’s Tai Chi for Arthritis) to be 1) a virtual experience (via Zoom) that includes a social component and 2) accessible and appropriate for those aging with mobility disabilities. We conducted the TechSAge Tele Tai Chi clinical trial to examine the effect of the intervention on physical activity and social connectedness (primary outcomes), as well as exercise self-efficacy, falls efficacy, depression, quality of life, and pain (secondary outcomes) for this population. The participant sample was community-dwelling adults (60-80 years of age) with a self-identified mobility disability (i.e., using a mobility aid or having serious difficulty walking or climbing stairs) for at least 10 years. The presentation will describe the rationale for the study, as well as the study design, methodology, and baseline data for the trial. We will also highlight the user-centered, iterative design approach we used in the design and evaluation of the intervention protocol.

